# Serious Complications of EUS-Guided Hepaticoesophagostomy due to Transmural Stent Migration

**DOI:** 10.1155/2021/4639286

**Published:** 2021-07-31

**Authors:** Mateusz Jagielski, Michał Zieliński, Jacek Piątkowski, Marek Jackowski

**Affiliations:** Department of General, Gastroenterological and Oncological Surgery, Collegium Medicum Nicolaus Copernicus University, Toruń, Poland

## Abstract

Thoracic complications, such as biliopleural fistula and bile leaking into the right pleural cavity, are serious adverse events of transmural endoscopic ultrasound- (EUS-) guided biliary drainage involving EUS-guided hepaticoesophagostomy (EUS-HES). In this article, the authors present endoscopic treatment of biliopleural fistula as a serious thoracic complication of EUS-HES. The authors highlight key components of EUS-guided transmural biliary drainage and their experience with particular emphasis on endoscopic treatment of thoracic complications.

## 1. Introduction

Transpapillary drainage of bile ducts during endoscopic retrograde cholangiopancreatography (ERCP) is a common and effective treatment for symptomatic patients with mechanical jaundice in the course of biliary obstruction [1–3]. When the drainage through duodenal papilla during ERCP fails, percutaneous transhepatic biliary drainage becomes the treatment of choice [1–6]. Therapeutic endoscopic ultrasound (EUS) techniques were developing intensively in the recent decades. If ERCP is unsuccessful or cannot be performed due to anatomical changes caused by the presence of neoplastic lesions or previous surgeries, EUS-guided transmural drainage of the biliary duct by establishing extra-anatomic anastomoses in the intrahepatic (hepaticoesophagostomy or hepaticogastrostomy) or extrahepatic (choledochoduodenostomy or cholecystoduodenostomy) biliary ducts provides an alternative method of treatment [1–6].

EUS-guided hepaticoesophagostomy is one of the least common methods of extra-anatomical biliary drainage [7]. In this paper, rare and serious complication of EUS-guided hepaticoesophagostomy was presented.

## 2. Case Presentation

**Figure 1:**
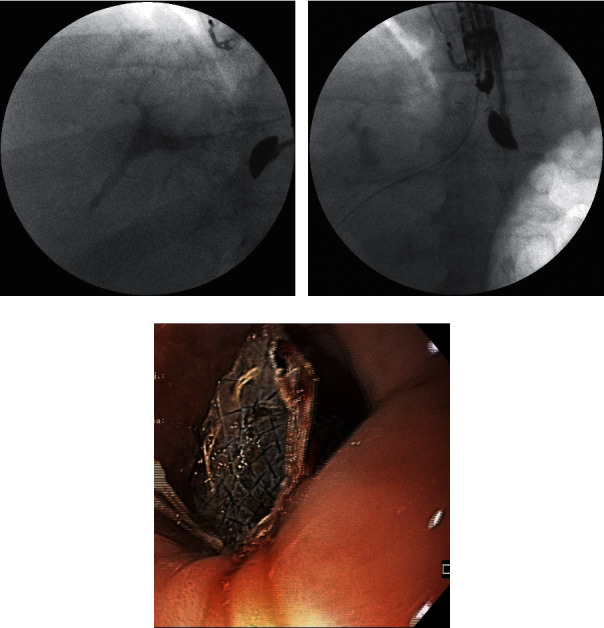
(a–c) EUS-guided hepaticoesophagostomy in a 57-year-old patient with an irresectable tumor of the pancreatic head and left hepatic lobe hypertrophy.

A 57-year-old male patient with malignant biliary obstruction caused by unresectable pancreatic head tumor (adenocarcinoma) was admitted to our medical center due to symptoms of obstructive jaundice. The patient was qualified for symptomatic endoscopic treatment. During ERCP, malignant infiltration of the duodenal wall in the peripapillary region preventing localization of the major duodenal papilla was stated. During the endoscopic examination, with no possibility of transpapillary palliative biliary drainage, the patient underwent transmural drainage of the biliary duct by establishing extra-anatomic anastomoses (Figures 1(a)‐1(c)). A linear array echoendoscope was introduced into the stomach, but due to left hepatic lobe hypertrophy in the endosonographic imaging, the echoendoscope was withdrawn into the esophagus. The dilated intrahepatic biliary ducts in the left lobe hepatic segments III were visible through the esophagus wall. Color Doppler ultrasound was used prior to performing an EUS-guided puncture through the esophagus wall to confirm the absence of vascular structures in the potential puncture line. The dilated biliary ducts in the left hepatic lobe were punctured using a 19G needle (EchoTip Ultra 19, Cook Medical, Bloomington, Indiana, USA) under endosonographic control. Following stylet removal, bile content aspiration was performed to confirm an intraductal needle tip location. Next, the contrast agent was administered via the intraductal needle under fluoroscopic control to obtain an antegrade cholangiogram (Figure 1(a)). After flushing the needle with saline solution, a rigid 0.035-inch guidewire (Dreamwire; Boston Scientific Corp., Marlborough, Massachusetts, USA) was introduced through the needle lumen into the bile duct. The guidewire was introduced into the left bile duct and then directed towards the common bile duct with the intention of gaining access to the duodenal lumen so as to continue the procedure using the rendezvous approach or to perform antegrade deployment of the transpapillary stent. Following several unsuccessful attempts to access the duodenum due to malignant stricture of the main bile duct or duodenum, the needle was withdrawn, while the position of the guidewire was maintained and fistula was established using a 10 Fr cystostome (Cook Endoscopy, Ireland). Half-coated self-expanding endoprosthesis (GIOBOR, diameter of 10 mm, length of 10 cm; Taewoong Medical, Gyeonggi-do, Korea) was introduced through the newly formed fistula under endosonographic and fluoroscopic guidance (Figures 1(b) and 1(c)). The catheter was then introduced through the endoprosthesis into the bile duct, and a contrast agent was administered for a follow-up cholangiographic examination to confirm correct positioning of the transmural endoprosthesis, correct biliary drainage, and the absence of any leaks from the biliary tract. Decrease of cholestasis parameters in blood tests was observed after the endoscopic procedure. The patient was discharged home in good general condition after three days.

**Figure 2:**
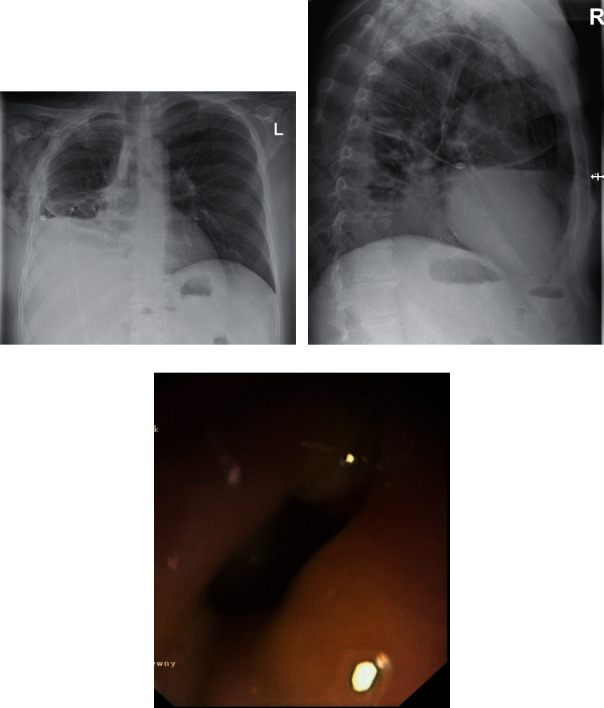
(a–c) Late complication of endoscopic treatment in the form of biliopleural fistula. Right pleural cavity drainage.

**Figure 3:**
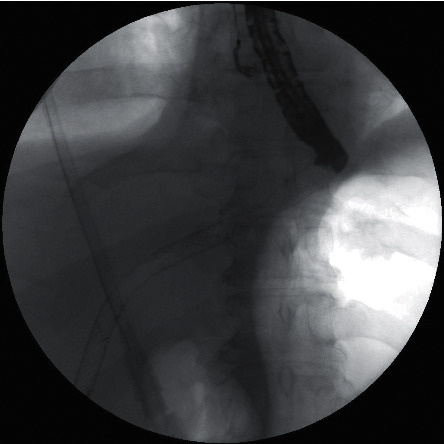
Endoscopic treatment of biliopleural fistula as a complication of endoscopic hepaticoesophagostomy

Twenty four days after the endoscopic procedure, the patient was admitted again to our department due to symptoms related to fluid in the right pleural cavity (Figures 2(a) and 2(b)). The clinical condition was dominated by dyspnoea. No signs of pleuritis were stated. In the physical examination, lowering of murmur over the right lung was stated. Basing on the clinical image and results of imaging examinations, the late complication of EUS-HES—transmural stent migration resulting in biliopleural fistula and bile leaking into the right pleural cavity—was diagnosed (Figures 2(a)–2(c)). The patient required drainage of the right pleural cavity (Figures 2(a) and 2(b)). This complication was successfully managed endoscopically (Figure 3). The distal end of the dislocated endoprosthesis was located in the biliary duct of the left hepatic lobe, but the proximal end of this stent was located in the right pleural cavity. During the endoscopic procedure, access through esophageal fistula to the dislocated stent was achieved under the control of the fluoroscopic image. Through the lumen of dislocated endoprosthesis, a rigid 0.035-inch guidewire (Dreamwire; Boston Scientific Corp., Marlborough, Massachusetts, USA) was introduced into biliary ducts. By the use of guidewire, across the dislocated transmural stent, another biliary self-expanding metal stent (using “nstent-in-stent” technique) with a diameter of 10 mm and length of 8 cm (Evolution® Biliary Controlled-Release Stent-Partially Covered, Cook Endoscopy, Ireland) was introduced and esophagobiliary anastomosis was splinted via the right pleural cavity (Figure 3). Seven days after the procedure, the patient was discharged home in good general condition without any symptoms to continue the oncological treatment. During the six-month follow-up, no complications were observed.

## 3. Discussion

EUS-guided extra-anatomical anastomoses of bile ducts to the gastrointestinal tract have been reported as an effective alternative to percutaneous biliary drainage, when ERCP is ineffective [1–9]. However, in some patients, transgastric or transduodenal approach to biliary ducts is not possible [7–9]. In these cases, EUS-guided hepaticoesophagostomy remains treatment of choice [7–9]. Nevertheless, EUS-guided hepaticoesophagostomy is the least known method of endoscopic extra-anatomical biliary anastomoses [7–9]. Only this year, the first case series of efficacy and safety of EUS-guided hepaticoesophagostomy was published [7]. Earlier on, the EUS-guided hepaticoesophagostomy procedure was being described as case reports [8, 9].

Rugivarodom et al. presented a retrospective study of the first case series of patients with malignant biliary obstruction who underwent EUS-guided hepaticoesophagostomy (EUS-HES) [7]. Technical success was achieved in 100%, and clinical success was achieved in 90.9% of the patients [7]. Treatment-related complications were reported in 27.27% of the patients (with no major procedure-related complications) [7]. Rugivarodom et al. described EUS-HES as a technically feasible and safe procedure for biliary drainage, especially in patients who have undergone left hepatic lobe hypertrophy [7]. The authors observed treatment complications in 3 out of 11 (27.27%) patients: bile leakage leading to mild peritonitis was observed in 1 patient and stent migration that was subsequently treated endoscopically was observed in 2 other patients [7]. No thoracic complications, such as pneumomediastinum or mediastinitis [7], which are serious complications of endoscopic treatment involving EUS-HES [8, 9], were observed by the authors in their study.

In case of lack of access to the extrahepatic biliary ducts, which is more preferred access in extra-anatomical endoscopic biliary duct anastomoses to the gastrointestinal tract, the intrahepatic access is performed (EUS-HES or EUS-HGS) [1–9]. In case when access to the intrahepatic ducts is possible, the gold standard is transgastric access in the form of EUS-HGS [1–9]. However, in some cases, when access to the intrahepatic ducts is not possible, especially in cases of advanced tumors with hypertrophy of left hepatic lobe, endoscopic anastomosis of intrahepatic ducts with esophagus (EUS-HES) may be a treatment of choice [7–9]. Both in EUS-HES and EUS-HGS, a special half-coated self-expanding endoprosthesis is located transmurally [7–9]. Uncoated half of the transmural stent is located intrahepatically, while the remaining part of the stent stays in the transmural anastomosis [7–9]. In our opinion, the EUS-HGS is a safer and more effective procedure than EUS-HES. Firstly, for EUS-HGS, dedicated half-coated self-expanding endoprosthesis is produced to reduce adverse events, for example, in the form of stent's migration, which is described here. There are no dedicated transmural stents for EUS-HES and stents for EUS-HGS are used during the EUS-HES procedure, which increases risk of complications. Secondly, EUS-HES is technically more demanding procedure than EUS-HGS, which is an additional adverse event's factor. Undoubtedly, EUS-HES requires higher technical skills of the endoscopist in the special experienced interventional endoscopic centers. Thirdly, during EUS-HES, distance between the bile ducts and gastrointestinal tract is usually bigger than during EUS-HGS, which is a result of anatomical conditions and severely increases the risk of stent migrations.

As it was stated above, complication of EUS-HES can be prevented by the use of specially designed transmural stents, which is the matter of the future. Additionally, EUS-HES procedures should be performed in the special experienced interventional endoscopic centers by an advanced endoscopist, which will increase the safety of the procedure. In our opinion, these are the only factors that may at the moment prevent described complication due to transmural stent migration.

Due to small amount of data concerning this procedure in current literature, complications and their management are very rarely available. For this reason, this publication is of substantive value because it shows endoscopic treatment of biliopleural fistula and bile leaking into the right pleural cavity as a rare complication of EUS-guided hepaticoesophagostomy.

## Data Availability

The data used to support the findings of this study are available from the corresponding author upon request.
